# Indirect sciatic nerve transection from gunshot induced comminuted femoral fracture

**DOI:** 10.1016/j.radcr.2026.01.056

**Published:** 2026-02-13

**Authors:** Laura E. Garton, Logan P. Haug, Keith T. Aziz, Rupert O. Stanborough, Daniel E. Wessell

**Affiliations:** a Department of Radiology, Mayo Clinic, 4500 San Pablo Road South, Jacksonville, FL, USA, 32224; b Department of Orthopedic Surgery, Mayo Clinic, 4500 San Pablo Road South, Jacksonville, FL, USA, 32224

**Keywords:** Sciatic nerve transection, Peripheral nerve injury, Comminuted fracture, Traumatic neuroma, Nerve allograft

## Abstract

Sciatic nerve transection is a severe peripheral nerve injury associated with profound motor and sensory deficits, often resulting in long-term disability if not promptly addressed. We report a case of persistent motor loss after a ballistic injury in a 31-year-old female, initially attributed to the concussive effect of the ballistic and later found to be the consequence of a bony fragment transection. One year after the initial trauma, radiographs at our institution demonstrated a displaced, posteriorly directed sharp bony fragment at the level of the distal femoral diaphysis. MRI revealed features consistent with a traumatic neuroma in discontinuity with fusiform enlargement of the terminal stump proximal to the bone fragment and an intraneural cyst of the stump distal to the fragment. This case underscores the importance of dedicated nerve imaging in temporal proximity to injury as early identification and surgical intervention is critical to maximize functional outcomes in complex ballistic nerve injuries.

## Introduction

Humans with peripheral nerve injury experience immediate loss of sensory and motor functions followed by Wallerian degeneration of the distal axonal segments over the next 3 to 7 days [1]. Axonal injuries can occur through compression, transection, repetitive friction, crushing, or stretching mechanisms [2]. Most peripheral nerves consist of nerve fascicles encapsulated by perineurium, surrounded by connective tissue and fat, bundled into an epineural sheath [3]. After injury, peripheral nerves have the remarkable capability to regenerate with a speed of 1 to 2 millimeters per day although junctional recovery may be poor if there is interval atrophy of muscle fibers or fibrosis [1]. Recovery is contingent upon preservation of the cell body, maintenance of neuromuscular junction signaling, and a milieu of factors including cytokines, growth factors, hormones, and the extracellular matrix [4,5]. Prompt recognition of peripheral nerve injury through dedicated imaging coupled with surgical intervention increases the probability of regeneration.

### Case

**Fig. 1:**
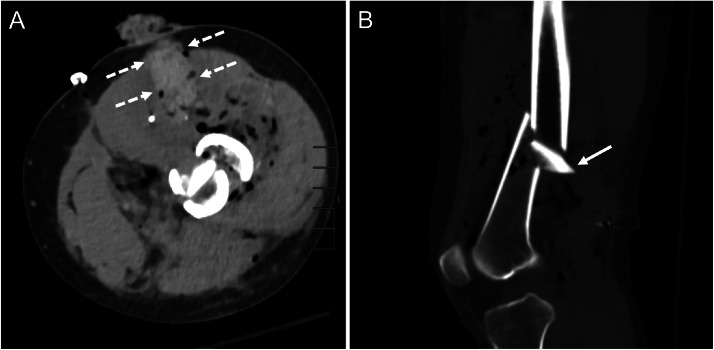
Axial (A) and sagittal (B) CT images of the left distal femur at initial presentation demonstrate the anteromedial to posterolateral trajectory (dashed arrows) of the projectile with extensive associated subcutaneous emphysema and a displaced comminuted distal femoral fracture with a posteriorly directed sharp bony fragment (arrow).

A 31-year-old female presented to our orthopedic surgery clinic for evaluation of left sciatic nerve injury after ballistic injury. Approximately 1 year prior to presentation, she sustained a gunshot wound to her distal anteromedial thigh resulting in a comminuted distal femoral fracture, popliteal artery dissection, and compartment syndrome (Fig. 1). She was initially treated at an outside hospital and underwent left retrograde femoral intramedullary nail placement, left popliteal artery dissection, and lower leg fasciotomies for compartment syndrome.

Immediately following the injury, the patient had no sensation or motor function to the lower leg. After 1 month of physical therapy, the patient reported partial return of sensation throughout her left lower extremity with residual decreased sensation to the lateral and plantar aspects of her left foot. She had persistent distal motor loss with inability to move her toes and ankle. Electromyography (EMG) 5 months after the initial injury was significant for severe axonal damage of the left sciatic nerve at the level of the knee with acute denervation. Although the patient experienced some return of sensation in her left lower extremity, there was no detectable motor or sensory signal in the left lower extremity below the level of the knee in the muscles innervated by the common peroneal and tibial nerves. Repeat EMG 1 year after the injury was again significant for absence of motor and sensory responses in the left lower extremity with no evidence of reinnervation to the distal leg muscles. On physical exam, the patient reported sensation to light touch in the saphenous, superficial peroneal, and deep peroneal nerve distributions. Sensation was subjectively diminished to the lateral plantar foot in both the sural and lateral plantar nerve distributions. Muscle strength was 0/5 in the tibialis anterior, extensor hallux longus, flexor hallux longus, gastrocnemius, peroneus brevis, and peroneus longus.

**Fig. 2:**
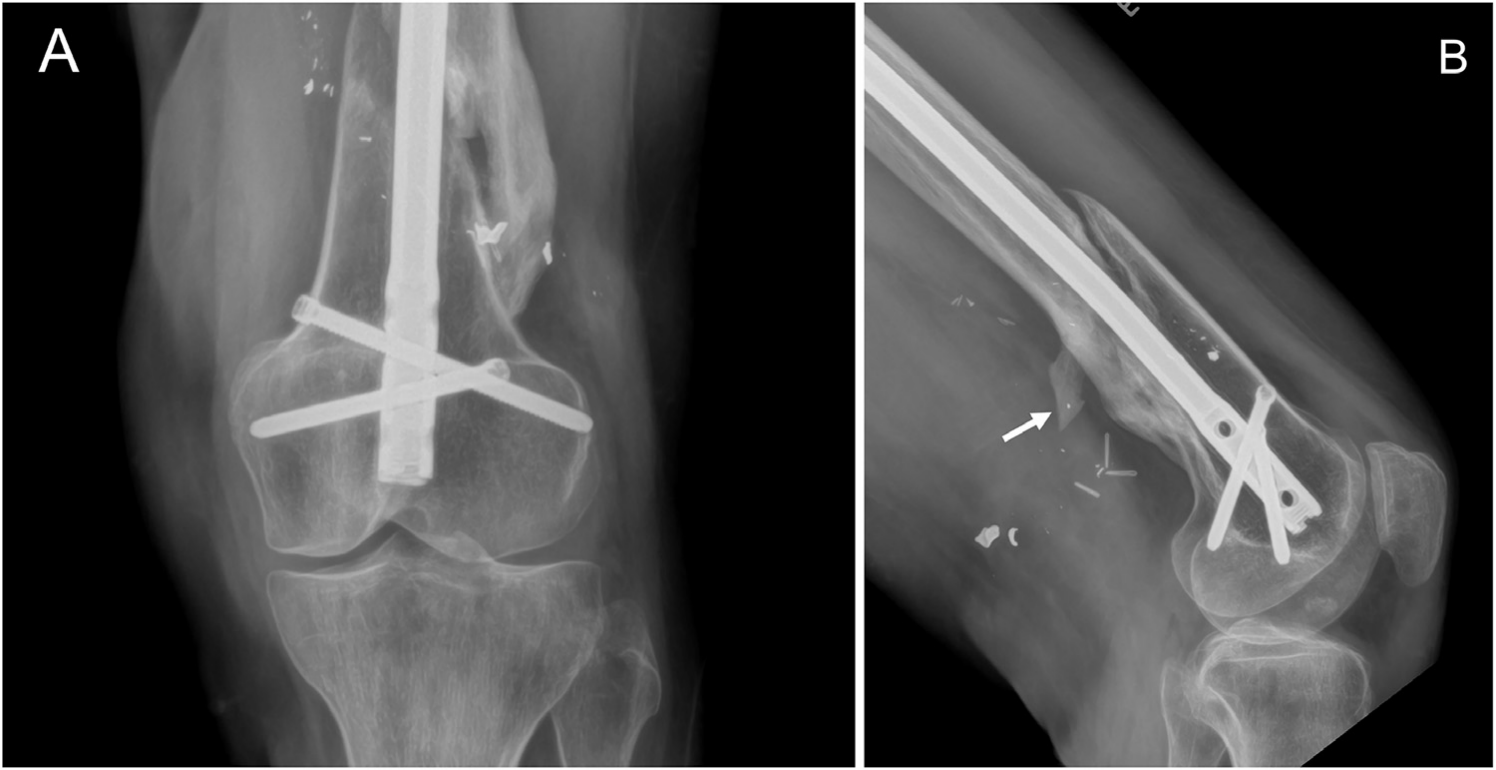
AP (A) and lateral (B) radiographs of the distal left femur 10 months after injury demonstrate improved fracture alignment and healing after placement of an intramedullary nail. The posteriorly directed fracture fragment remains unchanged in position after fixation (arrow) with surrounding metallic debris.

**Fig. 3:**
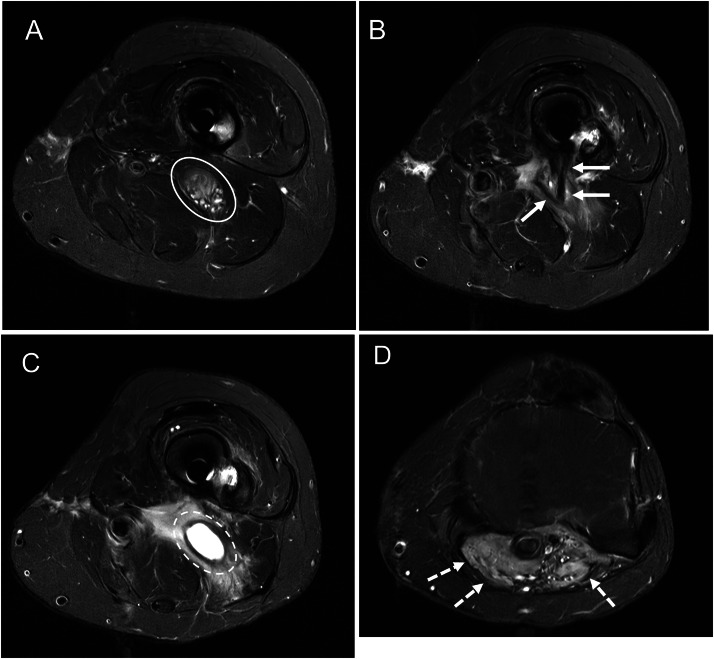
(A–C) Axial T2-weighted fat-saturated images of the distal femur show fusiform enlargement and fascicular expansion of the proximal stump of the transected sciatic nerve (white oval), consistent with traumatic neuroma. A posteriorly directed bone fragment with T2- hypointense cortical margins (arrows) transects the nerve just below this level. Immediately inferior to the bone fragment there is cystic dilation of the distal stump (dashed oval). (D) Axial T2-weighted fat-saturated image at the proximal tibia demonstrates diffuse edema within the gastrocnemius muscles (dashed arrows), indicative of denervation changes.

Radiographs at presentation to our hospital demonstrated interval intramedullary rod placement, metallic soft tissue debris, and a posteriorly directed sharp bone fragment (Fig. 2). Subsequent MRI was significant for fusiform enlargement of the sciatic nerve, consistent with a traumatic neuroma, just proximal to a posteriorly directed pointed bone fragment which completely transected the sciatic nerve (Fig. 3). Proximal and distal to the nerve stumps at the site of transection, the sciatic, tibial and peroneal nerves were normal in appearance.

**Fig. 4:**
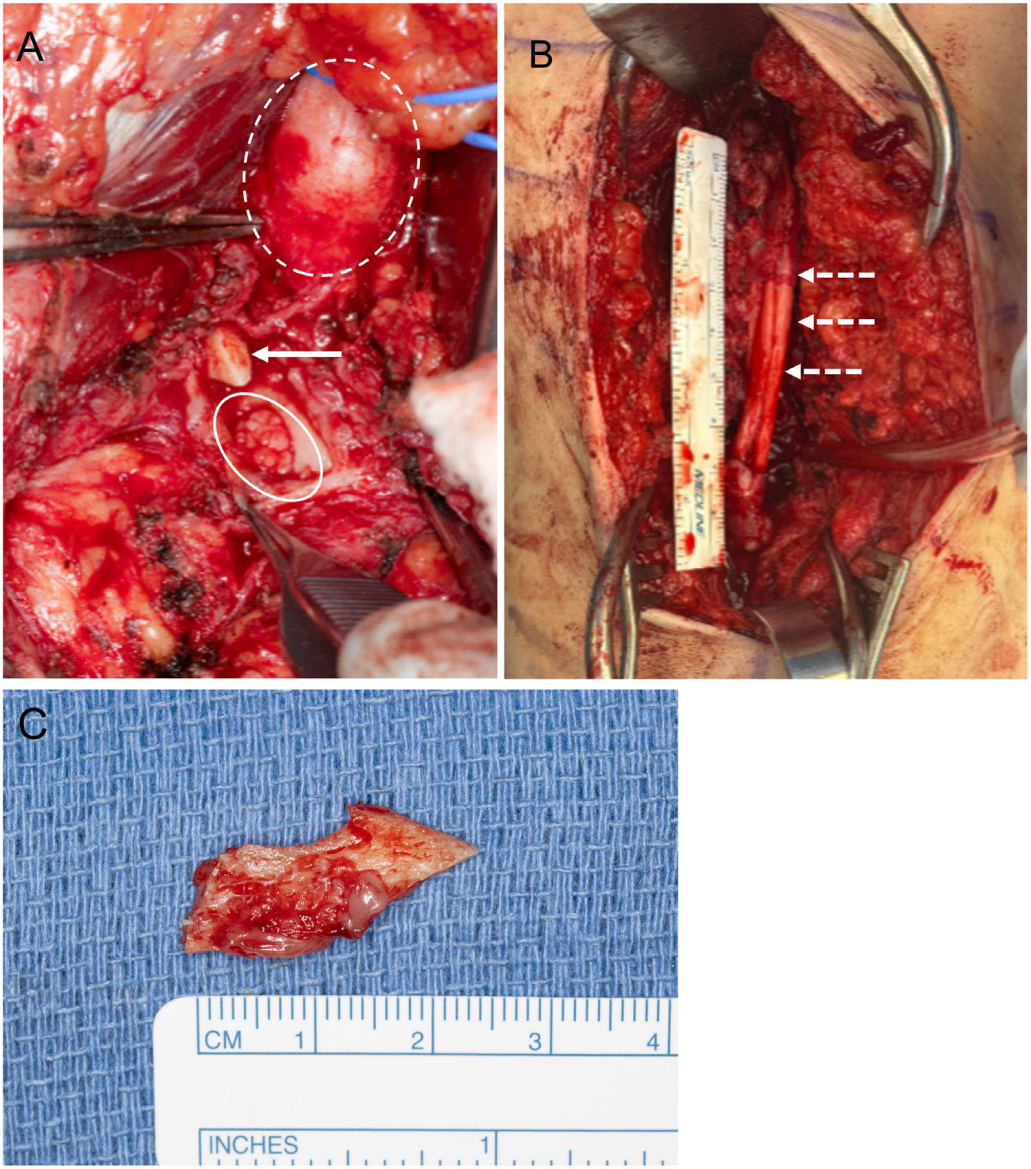
Intraoperative photograph before (A) sciatic nerve reconstruction demonstrates the distal stump with fascicular dilation (solid oval), the pointed edge of the posteriorly directed bone fragment (arrow), and cystic dilatation of the proximal stump (dashed oval). Photograph after reconstruction (B) shows the nerve allograft bridging the previously transected sciatic nerve (dashed arrows). Intraoperative photograph (C) demonstrates the bone fragment.

The patient elected to proceed with surgical repair and underwent left sciatic neurolysis, bone fragment excision, neuroma resection, and nerve allograft reconstruction (Fig. 4).

## Discussion

In our case, the patient experienced acute loss of motor and sensory function below the knee that was initially attributed to the concussive effect of the ballistic and was told to anticipate gradual return of function. The nerve injury went unrecognized both on initial CT lower extremity runoff at the time of injury and at the time of distal superficial femoral artery to below knee popliteal artery bypass. Identification of nerve injury on CT in the acute phase after injury can be difficult due to metallic debris, edema, and blood products which may efface the perineural fat planes obscuring nerves near the site of injury. This highlights the importance of dedicated nerve imaging in temporal proximity to injury when significant deficits are present, especially since a short interval between nerve injury and surgical repair is one of the strongest prognostic factors for functional recovery [[6], [7], [8]].

Neither in the acute nor subacute phase of injury was dedicated imaging of the sciatic nerve performed. We postulate that the providers may have been fearful of obtaining an MR due to radiofrequency (RF) induced heating and/or translational forces due to the extensive shrapnel and metallic debris present within the soft tissues from fragmentation of the projectile. However, Fountain et al. [9] has demonstrated that the presence of early projectile fragmentation prior to bone contact, a narrow debris trail, and incomplete projectile penetration, all of which were seen in our patient, are findings indicative of a nonferromagnetic composition. Projectiles that produce this pattern can safely be scanned under conditional settings without significant RF-induced heating or translational forces. However, in the absence of the aforementioned features, bullets should be presumed ferromagnetic until proven otherwise.

Prior reports of sciatic nerve transection/injury have been described, usually in the setting of projectile or blast related injuries but significantly displaced closed femoral fractures may also rarely result in sciatic nerve injury. Most case reports pertaining to sciatic nerve injury emphasize the clinical course and management rather than the imaging features. Additionally, sciatic nerve injury is often identified in the acute or subacute phase in contrast to our case. For instance, Al Zayer et al. [10] describe a case of sciatic nerve injury secondary to penetrating trauma identified approximately 3 weeks after injury due to bomb related blast injury. They describe identification of the injury sonographically but do not provide images. At surgical exploration nerve continuity was confirmed; however, small pieces of fragmented bone were found to have penetrated the sciatic nerve resulting in distal motor and sensory impairment. After resection of the bone fragments, the patient had significant functional recovery in the 6-month post-operative period based on sequential strength and sensation testing. Johnston et al. [11] describe a case of traumatic sciatic nerve transection in the context of a closed femoral shaft fracture identified within 1 week of injury after a motorcycle crash. This case parallels our own in that the severe osseous injury is likely the etiology of transection, and that MR imaging was performed to characterize the injury albeit in the acute phase.

Primary imaging modalities for evaluation of nerve injury include ultrasound (US) and MR. US is arguably the modality of choice for nerve injuries but is dependent on operator experience and does not allow for assessment of muscular denervation. Additionally, US may be limited by patient body habitus and nerve depth since beam penetration diminishes as soft tissue thickness between the probe and target nerve increases. Nerve injuries were originally classified by Herbert Seddon in 1943 into the following 3 types categories: (1) neuropraxia: demyelination but no anatomical nerve disruption, (2) axonotmesis: axonal loss with Wallerian degeneration but intact epineurium, perineurium, endoneurium, and (3) neurotmesis: complete nerve transection often with end-bulb neuromas [12]. In 1951, Sunderland expanded the classification to 5 distinct grades. Grades 1, 2, and 5 correspond to neuropraxia, axonotmesis and neurotmesis, respectively, while the newly added Grade 3 indicates endoneurial injury in conjunction with axonal loss and grade 4 indicates perineurial injury in addition to axonal loss and endoneurial injury. On MR imaging, these injuries have the following imaging features: Grade 1 - increased T2 signal with normal nerve caliber; Grade 2 - increased T2 signal with diffuse increase in caliber without focal enlargement; Grade 3 - increased T2 signal with focal fascicular enlargement; Grade 4 - focal nerve enlargement, signal heterogeneity, and fascicular transection with intact epineurium; Grade 5 - complete transection with a gap between the proximal and distal nerve stumps [12]. These features are also applicable to the sonographic evaluation of peripheral nerve injuries with the understanding that edema will manifest as hypoechogenicity.

Neurotmesis (Grade 5 injury), as shown in our case, is rarely a diagnostic dilemma and is readily characterized by complete nerve transection inclusive of the epineurium with or without an intervening nerve gap [12]. After traumatic peripheral nerve injuries, neuroma formation and denervation can occur. Grade 5 injuries may result in end-bulb type neuroma formation at the terminal nerve stumps. Stump neuromas are usually ovoid, T1 isointense and T2 hyperintense to muscle, and usually enhance. Their margins are often indistinct, and perineural fibrosis may be present [13]. After transection injuries, denervation is inevitable. In the acute phase, this presents as diffuse muscular edema with preserved muscular bulk. With time, the edema typically becomes less pronounced and heterogeneous with muscular atrophy and fatty metaplasia more dominant features.

Overall, in the setting of ballistic injury with significant functional nerve deficits, dedicated nerve imaging should be performed with US or MR during the acute or subacute phase of injury after patient stabilization to assess for nerve injury. Early identification of nerve injuries facilitates timely surgical intervention and maximizes the chance of functional recovery.

## Patient consent

Written, informed consent for publication of this case report was provided by the patient.
